# GPCR Heterodimerization in the Reproductive System: Functional Regulation and Implication for Biodiversity

**DOI:** 10.3389/fendo.2013.00100

**Published:** 2013-08-15

**Authors:** Honoo Satake, Shin Matsubara, Masato Aoyama, Tsuyoshi Kawada, Tsubasa Sakai

**Affiliations:** ^1^Suntory Foundation for Life Sciences, Bioorganic Research Institute, Osaka, Japan

**Keywords:** GPCR, heterodimer, reproduction, diversity hormones

## Abstract

A G protein-coupled receptor (GPCR) functions not only as a monomer or homodimer but also as a heterodimer with another GPCR. GPCR heterodimerization results in the modulation of the molecular functions of the GPCR protomer, including ligand binding affinity, signal transduction, and internalization. There has been a growing body of reports on heterodimerization of multiple GPCRs expressed in the reproductive system and the resultant functional modulation, suggesting that GPCR heterodimerization is closely associated with reproduction including the secretion of hormones and the growth and maturation of follicles and oocytes. Moreover, studies on heterodimerization among paralogs of gonadotropin-releasing hormone (GnRH) receptors of a protochordate, *Ciona intestinalis*, verified the species-specific regulation of the functions of GPCRs via multiple GnRH receptor pairs. These findings indicate that GPCR heterodimerization is also involved in creating biodiversity. In this review, we provide basic and current knowledge regarding GPCR heterodimers and their functional modulation, and explore the biological significance of GPCR heterodimerization.

## Introduction

The development of “omics” technologies and ensuring construction of a variety of databases provide vast information regarding primary sequences and functional domains of genes and proteins in diverse organisms, leading to annotation or prediction of biochemical and pharmacological propensities of novel genes and proteins. Even in this post-genomic era, several functions of proteins have yet to be fully elucidated or predicted. One of the most unpredictable and confounding post-translational protein functions is the heterodimerization of G protein-coupled receptors (GPCRs).

Currently, a wide range of GPCRs have been proved to function not only as monomers or homodimers but also as heterodimers formed after translation. It has been shown that GPCR heterodimerization alters or fine-tunes ligand binding, signaling, and internalization of GPCR protomers (1–9). The greatest difficulty in studies on GPCR heterodimers lies in the lack of procedures for the prediction of either GPCR protomer pairs for heterodimerization or the resultant functional alteration of GPCRs. Consequently, high-throughput analysis of GPCR heterodimers (e.g., “GPCR heterodimerome”) has not yet been accomplished. Despite this shortcoming, there have been increasing findings regarding the biological and pathological significance of GPCR heterodimerization.

**Figure 1:**
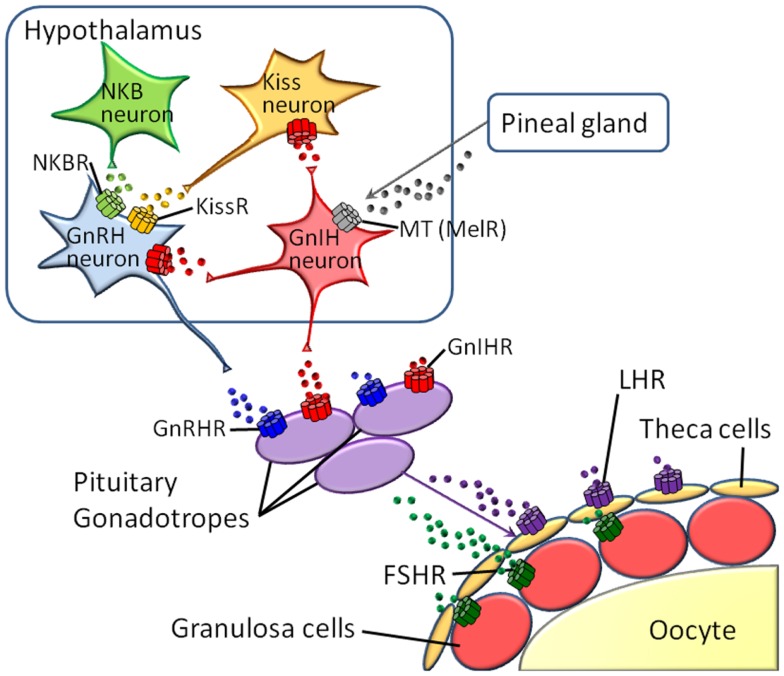
**Major GPCRs for neuropeptides or hormones in the hypothalamus-pituitary-gonad (HPG) axis**.

Reproduction is regulated by diverse neuropeptides and hormones, with the receptors belonging to the GPCR family, e.g., melatonin, kisspeptin, neurokinin B (NKB), gonadotropin-inhibitory hormone (GnIH), gonadotropin-releasing hormone (GnRH), luteinizing hormone (LH), follicle-stimulating hormone (FSH), and prostanoids (10–13). In vertebrates, these hormones and neuropeptides play crucial roles in the hypothalamus-pituitary-gonad (HPG) axis (Figure 1). Furthermore, various species-specific GPCRs for highly conserved cognate hormones or neuropeptides have been identified (14–18, Kawada et al., forthcoming). Collectively, these findings suggest that GPCR heterodimerization participates in the fine-tuning and diversification of reproductive functions. In this article, we provide an overview of GPCR heterodimerization and discuss the implication of GPCR heterodimers in reproductive functions and their diversification.

## GPCR Protomers as Allosteric Modulators

It is widely accepted that GPCRs can assume various active conformations which enable coupling with distinct G proteins and other associated proteins followed by particular signal transduction cascades (3, 8, 19, 20). Moreover, allosteric effectors interact with GPCRs at binding sites different from those for agonists or antagonists and modulate the conformations of GPCRs, leading to alterations in agonist/antagonist binding affinity or signal transduction (3, 8, 19, 20). Also of significance is that each of the active conformations responsible for individual signaling pathways is not interconvertible (3, 8, 19–21). Combined with experimental evidence that ligand binding and signaling of GPCR protomers are altered via heterodimerization, GPCR heterodimerization is believed to induce protomer-specific modulation (i.e., stabilization or instabilization) of active conformations as an endogenous allosteric modulator. This view is compatible with the fact that a single GPCR protomer acquires diverse biochemical and/or pharmacological properties via heterodimerization with different GPCR partners.

## Typical Functional Consequences of GPCR Heterodimerization

Obviously, colocalization of GPCR protomers in a cell is a prerequisite for the formation of the corresponding GPCR heterodimer in native tissues. However, many early studies demonstrated functional alteration of GPCRs only after co-transfection of cultured cells with two GPCRs but not at the level of endogenous co-expression in the same cells in a particular native tissue. Consequently, the biological and physiological significance of such “*in vitro*-only” GPCR heterodimers is highly questionable. Consistent with this, the International Union of Basic and Clinical Pharmacology (IUPHAR) release the paradigm for GPCR heterodimer studies in 2007 (2). First, interaction between GPCR protomers in native tissues should be proved by at least two different experimental procedures including co-immunoprecipitation, fluorescence resonance energy transfer (FRET), or bioluminescence resonance energy transfer (BRET). Second, alteration of biochemical or pharmacological functions of GPCRs should be observed in native tissues or co-transfected cells. Third, biological roles of GPCR heterodimers should be verified using gene-knockout or gene-silenced procedures. At present, meeting all of these criteria is too difficult. Thus, IUPHAR proposed that researchers fulfill at least two of the three criteria. In the following, we focus on GPCR heterodimers which were detected in native tissues (Table 1).

**Table 1:** **Typical functional alteration of GPCRs via heterodimerization**.

Heterodimer	Effect
GABAR_B1_–GABAR_B2_	Transition from ER to plasma membrane and function
T1R1–T1R3	Recognition of umami substances
T1R2–T1R3	Recognition of sweet substances
AT1–B2	Increase of IP_3_ accumulation induced by angiotensin II or bradykinin
MOR–DOR	Reduction in binding affinity of Met-enkephalin
	Increase in binding affinity of endomorphin-1 and Leu-enkephalin
	Shift of coupling of Gz to Gi
KOR–DOR	Enhancement of signaling induced by synthetic KOR agonists
OR1–CB1	Suppression of OR-triggered ERK phosphorylation by a CB1 antagonist
	Suppression of CB-triggered ERK phosphorylation by a OR1 antagonist
MC3R–GHSR	Increase in cAMP production induced by melanocortin
	Decrease in ghrelin-induced signaling
D1–D2	Shift of coupling of Gs to Gq/11
MT1–GPR50	Decrease of melatonin-binding, Gi-coupling/signaling, and internalization
NK1–MOR	Alternation of internalization and resensitization profile
R1–R4	Upregulation of ERK phosphorylation via Ca^2+^-dependent PKCα activation and Ca^2+^-independent PKCζ activation
R2–R4	Reduction in cAMP production via shift of coupling of Gs to Gi
EP1–β2AR	Dissociation of Gs from bA2R induced by EP1 agonists

*GABAR, GABA receptor; T1R, taste receptor; AT1, angiotensin receptor 1; B2 bradykinin receptor 2; MOR, μ-opioid receptor; DOR, δ-opioid receptor; KOR, κ-opioid receptor; OR1, orexin receptor 1; CB1, cannabinoid receptor 1; MC3R, melanocortin receptor 3; GHSR, ghrelin receptor; D1, dopamine receptor 1; MT1, melatonin receptor 1, NK1, tachykinin receptor 1; Ci-GnRHR, Ciona intestinalis GnRH receptor; EP1, prostaglandin E2 receptor 1*.

G protein-coupled receptor heterodimers are classified into two groups in light of their functions: obligatory and non-obligatory GPCR heterodimers. Obligatory GPCR heterodimers require heterodimerization of GPCR protomers to serve as functional receptors, such as gamma amino butyric acid (GABA) type B receptor and taste receptors. GABAR_B1_ alone is sequestered in the endoplasmic reticulum (ER) due to the presence of an ER retention signal, which is masked by heterodimerization with GABAR_B2_ (22–24). Moreover, the GABAR_B1_ protomer harbors a ligand-binding site, whereas the GABAR_B2_ protomer merely couples to G proteins (22–24). Therefore, the GABAR_B1_ -GABAR_B2_ heterodimer serves as an authentic GABA receptor. Taste receptors also exhibit heterodimerization-dependent pharmacological profiles. The heterodimer between T1R1 and T1R3 is exclusively responsive to umami taste, while the T1R2–T1R3 heterodimer is a specific receptor for sweet taste-inducing molecules (25–27).

In contrast, non-obligatory GPCR heterodimers are composed of the functional GPCR protomers and modulate the biochemical or pharmacological activities of the protomers (Table 1). Non-obligatory GPCR heterodimers account for the major population and exhibit diverse modulatory functions. In human embryonic kidney (HEK) 293 cells expressing the angiotensin II receptor (AT1)-bradykinin receptor (B2) heterodimer, angiotensin II triggered inositol triphosphate (IP_3_) accumulation much more potently and effectively than it did in the cells expressing AT1 alone, whereas IP_3_ accumulation by bradykinin was slightly weaker in cells expressing the AT1–B2 heterodimer than in the cells expressing only B2 (28). This enhancement was also detected *in vivo*, where AT1 and B2 were shown to form a heterodimer in smooth muscle, omental vessel, and platelets (28, 29).

The opioid receptor family is composed of three subtypes, namely, μ-, δ-, and κ-opioid receptors (MOR, DOR, and KOR), all of which mediate inhibition of cAMP production with different ligand-selectivity (30). Co-expression of MOR and DOR in HEK293 cells resulted in a 10-fold reduction in binding affinity of a synthetic MOR-selective agonist, DAMGO (31). Moreover, the MOR-DOR heterodimer differs in rank order of affinities for endogenous peptide ligands; Met-enkephalin, possessing the highest affinity for MOR among endogenous opioid peptides, exhibited twofold lower affinity to the MOR-DOR heterodimer, while the affinity of endomorphin-1 and Leu-enkephalin to the heterodimer was increased two to threefold, compared to MOR (31). Moreover, heterodimerization of MOR and DOR predominantly induced activation of a pertussis toxin-insensitive G protein, Gz in COS-7 cells, while monomeric or homodimeric MOR and DOR were coupled to a pertussis toxin-sensitive G protein, Gi (32). This is consistent with findings that the binding of ligands to the MOR–DOR heterodimer followed by signal transduction is resistant to pertussis toxin (32). A KOR-selective agonist, U69593, exhibited as potent and efficacious activities at the heterodimer as at KOR, whereas 6′-GNTI was a sixfold more potent agonist for the KOR–DOR heterodimer than for the KOR homodimer (33). More recently, *N*-naphthoyl-β-naltrex-amine (NNTA), a potent antagonist for MOR, was shown to manifest a prominent agonistic activity at MOR–DOR (34). In the mouse tail-flick assay, intrathecal NNTA elicited 100-fold greater antinociception, compared to intracerebroventricular administration (34). These heterodimerization-based pharmacological alterations are expected to provide crucial clues to understand why various *in vivo* pharmacological profiles are inconsistent with those from *in vitro* studies using cells expressing each opioid receptor alone and to develop more specific clinical agents for opioid receptors.

When an orexin receptor OR1 was co-expressed with a cannabinoid receptor CB1 in HEK293 cells, addition of a CB1-specific antagonist, SR-141716A, resulted in the suppression of orexin-triggered phosphorylation of ERK1/2 (35). Likewise, an OR1-specific antagonist, SB-674042, attenuated the ERK phosphorylation activated by a CB1 agonist, WIN55212-2 (35). These data verify the regulatory mechanism by which one GPCR protomer bound to an antagonist modulates the pharmacological profile of another GPCR protomer through heterodimerization.

Melanocortin receptor 3 (MC3R) and ghrelin receptor (GHSR) were found to be co-expressed in a number of neurons in the arcuate nucleus (36, 37). Co-transfection of MC3R and GHSR into COS-7 cells enhanced melanocortin-induced intracellular cAMP accumulation, compared with activation of MC3R in the absence of GHSR, whereas both agonist-independent basal and ghrelin-induced signaling of GHSR were diminished (36). These findings reveal mutual opposite signal modulation by each protomer and suggest that the molecular mechanism underlying a certain agonist-independent active conformation of a protomer is also involved in the regulation of the signaling functionalities of its partner GPCR in a heterodimer. Since MC3R and GHSR play pivotal roles in the orexigenic system, the MC3R–GHSR heterodimer is involved in hypothalamic body weight regulation.

There is increasing evidence for a pathological relevance of GPCR heterodimer. AT1–B2 heterodimer is highly likely to be functionally correlated with preeclampsia. The AT1–B2 heterodimer was more abundant on platelets of preeclamptic women than on platelets of normotensive pregnant women (29). Such increase in the number of heterodimers is due to enhanced expression of B2, as the expression level of B2 was elevated four to fivefold on platelets of preeclamptic women compared to platelets of normotensive pregnant women, whereas expression of AT1 was unchanged (29). Moreover, mobilization of intracellular calcium ions induced by angiotensin II was up-regulated 1.7- to 1.9-fold in platelets of preeclamptic women, compared to normotensive pregnant women (28, 29).

Heterodimerization between dopamine receptor subtypes, D1 and D2, has shown to be implicated in depression. The D1–D2 heterodimer was detected at higher levels in the post-mortem striatum of the patients compared to in normal subjects using co-immunoprecipitation and D1–D2 heteromer-selective antibodies (38). Moreover, dissociation of the D1–D2 heterodimer by an interfering peptide that disrupts the heteromer resulted in substantially reduced immobility in the forced swim test without affecting locomotor activity, and decreased escape failures in learned helplessness tests in rats (38). It should be noted that the heterodimerization between D1 and D2 leads to a drastic shift of G protein coupling; D1 and D2 monomer/homomer are coupled to Gs and Gi, respectively, while Gq/11 is a major G protein-coupled to the D1–D2 heterodimer (39).

More recently, MOR–DOR heterodimer was found to play pivotal roles in the opioid system. An interaction-disrupting peptide fragment for the MOR–DOR heterodimer enhanced morphine analgesia and reduced anti-nociceptive tolerance to morphine in mice (40).

## Heterodimers Among Reproduction – Associated GPCRs

### Melatonin receptor

Melatonin participates in reproductive functions via upregulation of the synthesis and secretion of GnIH in the hypothalamus of mammals and birds (10, 11). Moreover, melatonin receptors were also shown to be expressed in gonads (41), and melatonin significantly decreases testosterone secretion from LH/FSH-stimulated testes of European starlings before breeding (42). Two class A (rhodopsin-like) GPCRs for melatonin, MT1 and MT2, have been identified in mammals (1, 43). A human orphan GPCR, GPR50, sharing the highest sequence homology with MT1 and MT2, was shown to form a heterodimer with both receptors in HEK293 cells (1, 43). Moreover, heterodimerization of GPR50 with MT1 resulted in a marked reduction of the ability of MT1 to bind to ligands and to couple to G proteins, resulting in decreased in Gi protein-coupled intracellular signaling and β-arrestin – assisted internalization in HEK293 cells, whereas functions of MT2 were not affected (1, 43). These data indicate that GPR50 antagonizes MT1 but not MT2 via heterodimerization. In addition, this is the first report on the functional role of an orphan receptor as a protomer of a GPCR heterodimer.

### Tachykinin receptor

Tachykinins (TKs) are vertebrate and ascidian multifunctional brain/gut peptides involved in smooth muscle contraction, vasodilation, nociception, inflammation, neurodegeneration, and neuroprotection in a neuropeptidergic endocrine, paracrine fashion (44–48). The mammalian TK family consists of four major peptides: Substance P (SP), Neurokinin A (NKA), NKB, and Hemokinin-1/Endokinins (HK-1/EKs) (EK is a human homolog of mouse and rat HK-1). TK receptors belong to the class A GPCR family. Three subtypes of TK receptors, namely NK1, NK2, and NK3, have been identified in mammals, and several submammalian orthologs have been cloned or suggested by genomic database search. In the ascidian, *Ciona intestinalis*, authentic TK, and its cognate receptor, Ci-TK-I and Ci-TK-R were identified (49, 50). Recently, there are accumulating reports on reproductive roles of TKs as well as the expression of TKs and TK receptors in genital organs of mammals (46, 48, 49, 51–54). In *C. intestinalis*, Ci-TK-I enhances oocyte growth from the vitellogenic stage to the post-vitellogenic stage via upregulation of gene expression and enzymatic activity of several proteases such as cathepsin D, carboxypeptidase B1, and chymotrypsin (55–57). Over the past few years, there has been increasing evidence that NKB plays a central role in the direct enhancement of GnRH synthesis and release in the hypothalamus of mammals, eventually leading to the recognition of novel regulatory function in sexual maturation and reproduction [(58–65)].

Only one tachykinin receptor-relevant heterodimer has thus far been identified. NK1 and an opioid receptor subtype, MOR, were shown to co-exist in pain-processing brain regions, including trigeminal dorsal horn neurons, and to heterodimerize in co-transfected HEK293 cells (66). NK1–MOR heterodimerization altered their internalization and resensitization profile, while ligand binding and signaling intensities of the protomers were not affected. In cells expressing NK1–MOR heterodimer, both DAMGO and SP induced the recruitment of β-arrestin to the plasma membrane and internalization of NK1–MOR heterodimers with β-arrestin into the same endosomal compartment (66). Recent studies also verified that other tachykinin receptors, such as NK3, are co-localized with various GPCRs including kisspeptin receptors and opioid receptors (59). Consequently, tachykinin receptors are expected to form heterodimers with a wide variety of GPCRs, which, in turn, are potentially involved in the molecular mechanisms underlying novel reproductive functions.

### GnRH receptor

Gonadotropin-releasing hormones are hypothalamic decapeptides that regulate the HPG axis to control reproduction by releasing gonadotropins, FSH, and LH from the pituitary in vertebrates (Figure 1). The endogenous receptors, GnRHRs, which belong to the Class A GPCR family, have also been shown to possess species-specific paralogs forms in vertebrates. Type I GnRHRs, which completely lack a C-terminal tail region, are restricted to humans, rodents, and cows (14–16, 67, 68). Type II GnRHRs, which bear a C-terminal tail, are widely distributed throughout almost all vertebrates, whereas the type II *gnrhr* gene is silenced due to a deletion of functional domains or interruption of full-length translation by the presence of a stop codon in humans, chimpanzees, cows, and sheep (14–16, 67, 68). To date, no convincing evidence for heterodimerization of GnRHRs in native tissues has been provided.

Gonadotropin-releasing hormones have also been identified in a wide range of invertebrates that lack a pituitary (Kawada et al., forthcoming). To date, seven GnRH peptides (tGnRH-3 to -8 and Ci-GnRH-X) and four GnRH receptor subtypes (Ci-GnRHR-1 to -4) have been identified in *C. intestinalis* (69–71). Molecular phylogenetic analysis indicates that Ci-GnRHR2 (R2), R3, and R4 are *Ciona*-specific paralogs of R1 generated via gene duplication (70, 72). Only R1 activated IP_3_ generation followed by intracellular Ca^2+^ mobilization in response to tGnRH-6, whereas R2 and R3 exclusively stimulate cAMP production in response to multiple tGnRHs; tGnRH-6, -7, and -8 exhibited near-equipotent cAMP production via R2, which was 100-fold more potent than tGnRH-3, -4, and -5. tGnRH-3 and -5 specifically triggered R3-mediated cAMP production (70, 73–75). R4 is devoid of binding to any tGnRHs or of activating any signaling pathways (70). Recently, we have shown that the orphan paralog, R4, is responsible for the fine-tuning of the GnRHergic signaling via heterodimerization with R1. The R1–R4 heterodimer elicited a 10-fold more potent Ca^2+^ mobilization than R1 monomer/homodimer in a tGnRH-6-selective manner, while cAMP production by R1 was not modulated via heterodimerization with R4 (73). The R1–R4 heterodimer potentiated translocation of both Ca^2+^-dependent PKCα by tGnRH-6 and Ca^2+^-independent PKCζ by tGnRH-5 and -6, eventually leading to upregulation of ERK phosphorylation, compared with R1 alone (73). These results provide evidence that the species-specific GnRHR orphan paralog, R4, serves as an endogenous modulator for the fine-tuning of the activation of PKC subtype-selective signal transduction via heterodimerization with R1. R4 was also shown to heterodimerize with R2 specifically in test cells of vitellogenic oocytes (74). Of particular interest is that the R2–R4 heterodimer in HEK293 cells decreased cAMP production in a non-ligand selective manner via a shift from activation of Gs protein to Gi protein by R2, compared with R2 monomer/homodimer (74). Considering that R1–R4 elicited a 10-fold more potent Ca^2+^ mobilization than R1 monomer/homodimer in a ligand selective manner but did not affect cAMP production, these results indicate that R4 regulates differential GnRH signaling cascades via heterodimerization with R1 and R2 as an endogenous allosteric modulator. Collectively, these studies suggest that heterodimerization among GnRHR paralogs, including the species-specific orphan receptor subtype, is involved in rigorous and diversified GnRHergic signaling in a protochordate lacking an HPG axis.

### LH receptor

Luteinizing hormone is a central pituitary peptide hormone responsible for gonadal maturation (Figure 1). A single GPCR for LH has been identified in mammals. Although no LH receptor-containing GPCR heterodimer has been detected, studies on LH homodimers suggests that LH can also serve as a multifunctional protomer in various GPCR heterodimers. Co-expression of a ligand-binding-deficient LH receptor mutant and a signaling-deficient LH receptor mutant resulted in the restoration of normal gonadal and genital function in transgenic mice, indicating that LH receptor functions as a dimer *in vivo* (76).

### Prostaglandin receptor

Prostanoids consist of prostaglandin (PG) D, PGE_2_, PGF_2α_, PGI_2_, and thromboxane A_2_ and are responsible for a variety of actions in various tissues including the relaxation and contraction of various types of smooth muscles, pain transmission, fever generation, and sleep induction (77). Numerous studies have also proved that ovulation, corpus luteum development and regression are mediated by PGs (78–80). To date, eight GPCRs for PGs have been identified in mammals. Heterodimerization of a PGE_2_ receptor, EP1, with β2 adrenergic receptor (β2AR) caused considerable reduction in cAMP production by β2AR *via* enhancement of the dissociation of Gs protein from β2AR in the presence of endogenous or synthetic EP1 agonists in primary cultures of airway smooth muscle or COS-7 cells (81). Of importance in the functional regulation by the GPCR heterodimer is that EP1 have a direct inhibitory effects on bronchodilatory signaling but rather modulates the function of the β2AR. These findings strongly suggest that the heterodimerization of β2AR with EP1 causes the β2-agonist resistance found in asthma (81).

## Effects of GPCR Heterodimerization on Diversification of Animal Species and Biological Function

**Figure 2:**
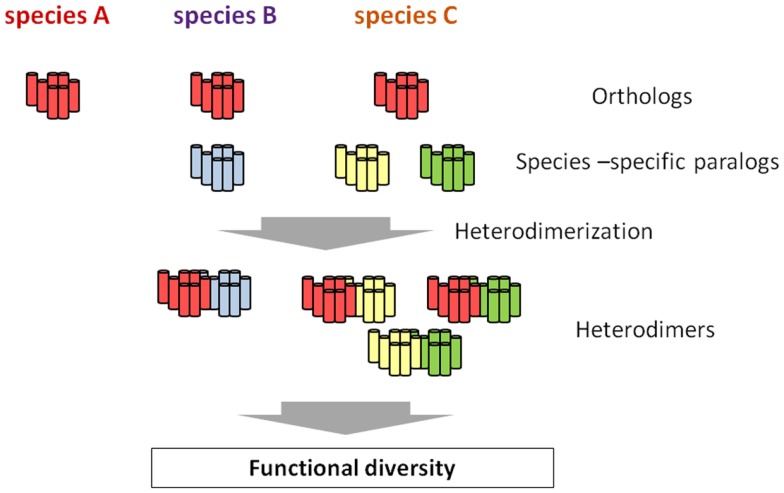
**Hypothetical scheme of the emergence of functional diversity *via* species-specific GPCR heterodimerization**. All the species conserve the authentic orthologous GPCR (red), whereas species B and C possess one or two additional species-specific paralogs, respectively (blue, yellow, and green). While no heterodimer involving the orthologous GPCR is formed, species-specific GPCR heterodimers occur in species B and C. such species-specific heterodimers are highly likely to be closely related functional diversity of a certain GPCR family, ultimately leading to the evolution and diversification of organisms to a large extent.

G protein-coupled receptors are largely categorized into two groups. The first group consists of GPCRs conserved as authentic “homologs” in various species, and the second one includes species-specific GPCRs. The latter is further classified into GPCRs for species-specific ligands and subtypes of GPCRs for highly conserved ligands in various species. For instance, *C. intestinalis* GnRH receptors consists of four GPCRs as stated above: R1, R2, R3, and R4. Phylogenetic tree and biochemical analyses proved that R1 is structurally and functionally homologous to vertebrate GnRH receptors, whereas R2, R3, and R4 are *C. intestinalis*-specific paralogs that occurred via gene duplication in the *Ciona* evolutionary lineage (70, Kawada et al., forthcoming). Likewise, species-specific GnRHR-III has been identified in teleost species (14, 15), and lamprey has also three GnRHRs which are genetically independent of teleost GnRHR subtypes (16). Such species-specific GPCR paralogs are thought to determine the functional diversity and physiological regulatory systems, because GPCR paralogs can form species-specific GPCR heterodimers, which, if expressed in the same cells, control the unique expansion and fine-tuning of GPCR-mediated signaling pathways (Figure 2), as shown for *C. intestinalis* GnRHRs (73, 74). In other words, heterodimerization involving species-specific GPCRs is highly likely to contribute to the evolution and diversification of organisms to a large extent.

## Conclusion and Perspectives

To date, GPCR heterodimerization has attracted keen attentions in light of biochemical and pharmacological features of GPCRs and the development of drugs with high selectivity, given that GPCR heterodimerization has been explored almost exclusively in mammals, except for *C. intestinalis* GnRHRs. Nevertheless, recent studies in various fields suggest that GPCR heterodimerization plays crucial roles in the regulation of the HPG axis and the evolution and diversification of reproductive functions. In this regard, of special interest is whether kisspeptin receptors or GnIH receptors heterodimerize with any GPCRs. Moreover, heterodimerization involving species-specific GPCR paralogs is expected to be responsible for the emergence of unique physiological functions in the respective organisms. Accordingly, combined with the fact that GPCRs form corresponding heterodimers after translation, investigation of GPCR heterodimerization in non-mammalian organisms will provide novel insight into the generation of biodiversity directed by a post-translational protein interaction.

In keeping with this issue, of particular interest is the clarification of the *in vivo* functional correlation between GPCR heterodimerization and biological events. Real-time imaging of GPCR heterodimerization could enable the visualization of biological functions of GPCR heterodimers of interest. Although this experimental strategy is unlikely to be applied to mammals, organisms equipped with transparent or semi-transparent skins, including ascidian, medaka, or zebrafish, are useful for live-imaging of GPCR heterodimers (50). Such studies are currently in progress in our laboratory.

## Conflict of Interest Statement

The authors declare that the research was conducted in the absence of any commercial or financial relationships that could be construed as a potential conflict of interest.

## References

[1] Levoye A Dam J Ayoub MA Guillaume JL Couturier C Delagrange P The orphan GPR50 receptor specifically inhibits MT1 melatonin receptor function through heterodimerization. EMBO J (2006) 25:3012–23 10.1038/sj.emboj.7601193 16778767 PMC1500982

[2] Pin JP Neubig R Bouvier M Devi L Filizola M Javitch JA International Union of Basic and Clinical Pharmacology. LXVII. Recommendations for the recognition and nomenclature of G protein-coupled receptor heteromultimers. Pharmacol Rev (2007) 59:5–13 10.1124/pr.59.1.5 17329545

[3] Satake H Sakai T Recent advances and perceptions in studies of heterodimerization between G protein-coupled receptors. Protein Pept Lett (2008) 15:300–8 10.2174/092986608783744207 18336362

[4] Milligan G G protein-coupled receptor hetero-dimerization: contribution to pharmacology and function. Br J Pharmacol (2009) 158:5–14 10.1111/j.1476-5381.2009.00169.x 19309353 PMC2795239

[5] Del Burgo LS Milligan G Heterodimerisation of G protein-coupled receptors: implications for drug design and ligand screening. Expert Opin Drug Discov (2010) 5:461–74 10.1517/17460441003720467 22823130

[6] Kamal M Jockers R Biological significance of GPCR heteromerization in the neuro-endocrine system. Front Endocrinol (Lausanne) (2011) 2:2 10.3389/fendo.2011.00002 22649357 PMC3355952

[7] Tadagaki K Jockers R Kamal M History and biological significance of GPCR heteromerization in the neuroendocrine system. Neuroendocrinology (2012) 95:223–31 10.1159/000330000 22156565

[8] Goupil E Laporte SA Hébert TE Functional selectivity in GPCR signaling: understanding the full spectrum of receptor conformations. Mini Rev Med Chem (2012) 12:817–30 10.2174/138955712800959143 22681252

[9] Borroto-Escuela DO Romero-Fernandez W Garriga P Ciruela F Narvaez M Tarakanov AO G protein-coupled receptor heterodimerization in the brain. Methods Enzymol (2013) 521:281–94 10.1016/B978-0-12-391862-8.00015-6 23351745

[10] Tsutsui K Ubuka T Bentley GE Kriegsfeld LJ Gonadotropin-inhibitory hormone (GnIH): discovery, progress and prospect. Gen Comp Endocrinol (2012) 177:305–14 10.1016/j.ygcen.2012.02.013 22391238 PMC3378827

[11] Ubuka T Son YL Tobari Y Tsutsui K Gonadotropin-inhibitory hormone action in the brain and pituitary. Front Endocrinol (Lausanne) (2012) 3:148 10.3389/fendo.2012.00148 23233850 PMC3515997

[12] Christensen A Bentley GE Cabrera R Ortega HH Perfito N Wu TJ Hormonal regulation of female reproduction. Horm Metab Res (2012) 44:587–91 10.1055/s-0032-1306301 22438212 PMC3647363

[13] Franceschini I Desroziers E Development and aging of the kisspeptin-GPR54 system in the mammalian brain: what are the impacts on female reproductive function? Front Endocrinol (Lausanne) (2013) 4:22 10.3389/fendo.2013.00022 23543285 PMC3610010

[14] Okubo K Nagahama Y Structural and functional evolution of gonadotropin-releasing hormone in vertebrates. Acta Physiol (Oxf) (2008) 193:3–15 10.1111/j.1748-1716.2008.01832.x 18284378

[15] Lindemans M Janssen T Beets I Temmerman L Meelkop E Schoofs L Gonadotropin-releasing hormone and adipokinetic hormone signaling systems share a common evolutionary origin. Front Endocrinol (Lausanne) (2011) 2:16 10.3389/fendo.2011.00016 22649364 PMC3356000

[16] Sower SA Decatur WA Joseph NT Freamat M Evolution of vertebrate GnRH receptors from the perspective of a basal vertebrate. Front Endocrinol (Lausanne) (2012) 3:140 10.3389/fendo.2012.00140 23181055 PMC3500703

[17] Kanda S Oka Y Structure, synthesis, and phylogeny of kisspeptin and its receptor. Adv Exp Med Biol (2013) 784:9–26 10.1007/978-1-4614-6199-9_2 23550000

[18] Gopurappilly R Ogawa S Parhar IS Functional significance of GnRH and kisspeptin, and their cognate receptors in teleost reproduction. Front Endocrinol (Lausanne) (2013) 4:24 10.3389/fendo.2013.00024 23482509 PMC3591744

[19] Fanelli F De Benedetti PG Update 1 of: computational modeling approaches to structure-function analysis of G protein-coupled receptors. Chem Rev (2011) 111:R438–535 10.1021/cr100437t 22165845

[20] Fuxe K Borroto-Escuela DO Marcellino D Romero-Fernandez W Frankowska M Guidolin D GPCR heteromers and their allosteric receptor-receptor interactions. Curr Med Chem (2012) 19:356–63 10.2174/092986712803414259 22335512

[21] Milligan G Smith NJ Allosteric modulation of heterodimeric G-protein-coupled receptors. Trends Pharmacol Sci (2007) 12:615–20 10.1016/j.tips.2007.11.001 18022255

[22] Kaupmann K Malitschek B Schuler V Heid J Froestl W Beck P GABA(B)-receptor subtypes assemble into functional heteromeric complexes. Nature (1998) 396:683–7 10.1038/25360 9872317

[23] Duthey B Caudron S Perroy J Bettler B Fagni L Pin JP A single subunit (GB2) is required for G-protein activation by the heterodimeric GABA(B) receptor. J Biol Chem (2002) 277:3236–41 10.1074/jbc.M108900200 11711539 PMC2566549

[24] Pin JP Kniazeff J Liu J Binet V Goudet C Rondard P Allosteric functioning of dimeric class C G-protein-coupled receptors. FEBS J (2005) 272:2947–55 10.1111/j.1742-4658.2005.04728.x 15955055

[25] Nelson G Hoon MA Chandrashekar J Zhang Y Ryba NJ Zuker CS Mammalian sweet taste receptors. Cell (2001) 106:381–90 10.1016/S0092-8674(01)00451-2 11509186

[26] Nelson G Chandrashekar J Hoon MA Feng L Zhao G Ryba NJ An amino-acid taste receptor. Nature (2002) 416:199–202 10.1038/nature726 11894099

[27] Xu H Staszewski L Tang H Adler E Zoller M Li X Human receptors for sweet and umami taste. Proc Natl Acad Sci USA (2004) 101:14258–63 10.1073/pnas.0404384101 15353592 PMC521102

[28] AbdAlla S Lother H Quitterer U AT1-receptor heterodimers show enhanced G-protein activation and altered receptor sequestration. Nature (2000) 407:94–8 10.1038/35024095 10993080

[29] AbdAlla S Lother H el Massiery A Quitterer U Increased AT(1) receptor heterodimers in preeclampsia mediate enhanced angiotensin II responsiveness. Nat Med (2001) 7:1003–9 10.1038/nm0901-1003 11533702

[30] Kieffer BL Opioids: first lessons from knockout mice. Trends Pharmacol Sci (1999) 20:19–26 10.1016/S0165-6147(98)01279-6 10101958

[31] Gomes I Jordan BA Gupta A Trapaidze N Nagy V Devi LA Heterodimerization of mu and delta opioid receptors: a role in opiate synergy. J Neurosci (2000) 20:RC110 11069979 10.1523/JNEUROSCI.20-22-j0007.2000 PMC3125672

[32] Fan T Varghese G Nguyen T Tse R O’Dowd BF George SR A role for the distal carboxyl tails in generating the novel pharmacology and G protein activation profile of mu and delta opioid receptor hetero-oligomers. J Biol Chem (2005) 280:38478–88 10.1074/jbc.M505644200 16159882

[33] Waldhoer M Fong J Jones RM Lunzer MM Sharma SK Kostenis E A heterodimer-selective agonist shows in vivo relevance of G protein-coupled receptor dimers. Proc Natl Acad Sci USA (2005) 102:9050–5 10.1073/pnas.0501112102 15932946 PMC1157030

[34] Yekkirala AS Lunzer MM McCurdy CR Powers MD Kalyuzhny AE Roerig SC N-naphthoyl-beta-naltrexamine (NNTA), a highly selective and potent activator of μ/kappa-opioid heteromers. Proc Natl Acad Sci USA (2011) 108:5098–103 10.1073/pnas.1016277108 21385944 PMC3064379

[35] Ellis J Pediani JD Canals M Milasta S Milligan G Orexin-1receptor-cannabinoid CB1 receptor heterodimerization results in both ligand-dependent and -independent coordinated alterations of receptor localization and function. J Biol Chem (2006) 281:38812–24 10.1074/jbc.M602494200 17015451

[36] Rediger A Piechowski CL Yi CX Tarnow P Strotmann R Grüters A Mutually opposite signal modulation by hypothalamic heterodimerization of ghrelin and melanocortin-3 receptors. J Biol Chem (2011) 286:39623–31 10.1074/jbc.M111.287607 21940628 PMC3234785

[37] Rediger A Piechowski CL Habegger K Grüters A Krude H Tschöp MH MC4R dimerization in the paraventricular nucleus and GHSR/MC3R heterodimerization in the arcuate nucleus: is there relevance for body weight regulation? Neuroendocrinology (2012) 9:277–88 10.1159/000334903 22327910

[38] Pei L Li S Wang M Diwan M Anisman H Fletcher PJ Uncoupling the dopamine D1-D2 receptor complex exerts antidepressant-like effects. Nat Med (2010) 16:1393–5 10.1038/nm.2263 21113156

[39] Rashid AJ So CH Kong MM Furtak T El-Ghundi M Cheng R D1-D2 dopamine receptor heterooligomers with unique pharmacology are coupled to rapid activation of Gq/11 in the striatum. Proc Natl Acad Sci USA (2007) 104:654–9 10.1073/pnas.0604049104 17194762 PMC1766439

[40] He SQ Zhang ZN Guan JS Liu HR Zhao B Wang HB Facilitation of μ-opioid receptor activity by preventing δ-opioid receptor-mediated codegradation. Neuron (2011) 69:120–31 10.1016/j.neuron.2010.12.001 21220103

[41] McGuire NL Bentley GE Neuropeptides in the gonads: from evolution to pharmacology. Front Pharmacol (2010) 1:114 10.3389/fphar.2010.00114 21607065 PMC3095369

[42] McGuire NL Kangas K Bentley GE Effects of melatonin on peripheral reproductive function: regulation of testicular GnIH and testosterone. Endocrinology (2011) 152:3461–70 10.1210/en.2011-1053 21771888

[43] Levoye A Dam J Ayoub MA Guillaume JL Jockers R Do orphan G-protein-coupled receptors have ligand-independent functions? New insights from receptor heterodimers. EMBO Rep (2006) 7:1094–8 10.1038/sj.embor.7400838 17077864 PMC1679777

[44] Severini C Improta G Falconieri-Erspamer G Salvadori S Erspamer V The tachykinin peptide family. Pharmacol Rev (2002) 54:285–322 12037144 10.1124/pr.54.2.285

[45] Almeida TA Rojo J Nieto PM Pinto FM Hernandez M Martin JD Tachykinins and tachykinin receptors: structure and activity relationships. Curr Med Chem (2005) 11:2045–81 10.2174/0929867043364748 15279567

[46] Satake H Kawada T Overview of the primary structure, tissue-distribution, and functions of tachykinins and their receptors. Curr Drug Targets (2006) 7:963–74 10.2174/138945006778019273 16918325

[47] Page NM Characterization of the gene structures, precursor processing and pharmacology of the endokinin peptides. Vascul Pharmacol (2006) 45:200–8 10.1016/j.vph.2005.08.028 16931167

[48] Satake H Aoyama M Sekiguchi T Kawada T Insight into molecular and functional diversity of tachykinins and their receptors. Protein Pept Lett (2013) 20:615–27 10.2174/0929866511320060002 22630127

[49] Satake H Ogasawara M Kawada T Masuda K Aoyama M Minakata H Tachykinin and tachykinin receptor of an ascidian, *Ciona intestinalis*: evolutionary origin of the vertebrate tachykinin family. J Biol Chem (2004) 279:53798–805 10.1074/jbc.M408161200 15485888

[50] Satake H Kawada T Aoyama M Sekiguchi T Sakai T Ascidians: new model orgnanisms for reproductive endocrinology. In: Aimaretti G Marzullo P Prodam F editors. Update on Mechanisms of Hormone Action – Focus on Metabolism, Growth and Reproductions. Vienna: IN-TECH (2011). p. 313–36

[51] Pennefather JN Patak E Ziccone S Lilley A Pinto FM Page NM Regulation of the stimulant actions of neurokinin a and human hemokinin-1 on the human uterus: a comparison with histamine. Biol Reprod (2006) 75:334–41 10.1095/biolreprod.106.051508 16707771

[52] Ravina CG Seda M Pinto FM Orea A Fernandez-Sanchez M Pintado CO A role for tachykinins in the regulation of human sperm motility. Hum Reprod (2007) 22:1617–25 10.1093/humrep/dem069 17437961

[53] Patak E Pennefather JN Gozali M Candenas ML Kerr K Exintaris B Functional characterisation of hemokinin-1 in mouse uterus. Eur J Pharmacol (2008) 601:148–53 10.1016/j.ejphar.2008.10.036 18977217

[54] Pinto FM Ravina CG Subiran N Cejudo-Román A Fernández-Sánchez M Irazusta J Autocrine regulation of human sperm motility by tachykinins. Reprod Biol Endocrinol (2010) 8:104 10.1186/1477-7827-8-104 20796280 PMC2936315

[55] Aoyama M Kawada T Fujie M Hotta K Sakai T Sekiguchi T A novel biological role of tachykinins as an up-regulator of oocyte growth: identification of an evolutionary origin of tachykininergic functions in the ovary of the ascidian, *Ciona intestinalis*. Endocrinology (2008) 149:4346–56 10.1210/en.2008-0323 18483149

[56] Aoyama M Kawada T Satake H Localization and enzymatic activity profiles of the proteases responsible for tachykinin-directed oocyte growth in the protochordate, *Ciona intestinalis*. Peptides (2012) 34:186–92 10.1016/j.peptides.2011.07.019 21827805

[57] Kawada T Ogasawara M Sekiguchi T Aoyama M Hotta K Oka K Peptidomic analysis of the central nervous system of the protochordate, *Ciona intestinalis*: homologs and prototypes of vertebrate peptides and novel peptides. Endocrinology (2011) 152:2416–27 10.1210/en.2010-1348 21467196

[58] Topaloglu AK Reimann F Guclu M Yalin AS Kotan LD Porter KM TAC3 and TACR3 mutations in familial hypogonadotropic hypogonadism reveal a key role for Neurokinin B in the central control of reproduction. Nat Genet (2009) 41:354–8 10.1038/ng.306 19079066 PMC4312696

[59] Rance NE Krajewski SJ Smith MA Cholanian M Dacks PA Neurokinin B and the hypothalamic regulation of reproduction. Brain Res (2010) 1364:116–28 10.1016/j.brainres.2010.08.059 20800582 PMC2992576

[60] Lasaga M Debeljuk L Tachykinins and the hypothalamo-pituitary-gonadal axis: an update. Peptides (2011) 32:1972–8 10.1016/j.peptides.2011.07.009 21801774

[61] Molnár CS Vida B Sipos MT Ciofi P Borsay BÁ Rácz K Morphological evidence for enhanced kisspeptin and neurokinin B signaling in the infundibular nucleus of the aging man. Endocrinology (2012) 153:5428–39 10.1210/en.2012-1739 23011920 PMC3473202

[62] Grachev P Li XF Kinsey-Jones JS di Domenico AL Millar RP Lightman SL Suppression of the GnRH pulse generator by neurokinin B involves a κ-opioid receptor-dependent mechanism. Endocrinology (2012) 153:4894–904 10.1210/en.2012-1574 22903614

[63] Hrabovszky E Sipos MT Molnár CS Ciofi P Borsay BÁ Gergely P Low degree of overlap between kisspeptin, neurokinin B, and dynorphin immunoreactivities in the infundibular nucleus of young male human subjects challenges the KNDy neuron concept. Endocrinology (2012) 153:4978–89 10.1210/en.2012-1545 22903610 PMC3512020

[64] Gill JC Navarro VM Kwong C Noel SD Martin C Xu S Increased neurokinin B (Tac2) expression in the mouse arcuate nucleus is an early marker of pubertal onset with differential sensitivity to sex steroid-negative feedback than Kiss1. Endocrinology (2012) 153:4883–93 10.1210/en.2012-1529 22893725 PMC3512019

[65] Ruiz-Pino F Navarro VM Bentsen AH Garcia-Galiano D Sanchez-Garrido MA Ciofi P Neurokinin B and the control of the gonadotropic axis in the rat: developmental changes, sexual dimorphism, and regulation by gonadal steroids. Endocrinology (2012) 153:4818–29 10.1210/en.2012-1287 22822161 PMC3512006

[66] Pfeiffer M Kirscht S Stumm R Koch T Wu D Laugsch M Heterodimerization of substance P and mu-opioid receptors regulates receptor trafficking and resensitization. J Biol Chem (2003) 278:51630–7 10.1074/jbc.M307095200 14532289

[67] Kah O Lethimonier C Somoza G Guilgur LG Vaillant C Lareyre JJ GnRH and GnRH receptors in metazoa: a historical, comparative, and evolutive perspective. Gen Comp Endocrinol (2007) 153:346–64 10.1016/j.ygcen.2007.01.030 17350014

[68] Millar RP Pawson AJ Morgan K Rissman EF Lu ZL Diversity of actions of GnRHs mediated by ligand-induced selective signaling. Front Neuroendocrinol (2008) 29:17–35 10.1016/j.yfrne.2007.06.002 17976709 PMC2667102

[69] Adams BA Tello J Erchegyi J Warby C Hong DJ Akinsanya KO Six novel gonadotropin-releasing hormones are encoded as triplets on each of two genes in the protochordate, *Ciona intestinalis*. Endocrinology (2003) 144:1907–19 10.1210/en.2002-0216 12697698

[70] Tello JA River J Sherwood NM Tunicate gonadotropin-releasing hormone (GnRH) peptides selectively activate *Ciona intestinalis* GnRH receptors and the green monkey type II GnRH receptor. Endocrinology (2005) 146:4061–73 10.1210/en.2004-1558 15961566

[71] Kawada T Aoyama M Okada I Sakai T Sekiguchi T Ogasawara M A novel inhibitory gonadotropin-releasing hormone-related neuropeptide in the ascidian, *Ciona intestinalis*. Peptides (2009) 30:2200–5 10.1016/j.peptides.2009.08.014 19712719

[72] Kusakabe T Mishima S Shimada I Kitajima Y Tsuda M Structure, expression, and cluster organization of genes encoding gonadotropin-releasing hormone receptors found in the neural complex of the ascidian *Ciona intestinalis*. Gene (2003) 322:77–84 10.1016/j.gene.2003.08.013 14644499

[73] Sakai T Aoyama M Kusakabe T Tsuda M Satake H Functional diversity of signaling pathways through G protein-coupled receptor heterodimerization with a species-specific orphan receptor subtype. Mol Biol Evol (2010) 27:1097–106 10.1093/molbev/msp319 20026483

[74] Sakai T Aoyama M Kawada T Kusakabe T Tsuda M Satake H Evidence for differential regulation of GnRH signaling via heterodimerization among GnRH receptor paralogs in the protochordate, *Ciona intestinalis*. Endocrinology (2012) 153:1841–9 10.1210/en.2011-1668 22294747

[75] Kusakabe TG Sakai T Aoyama M Kitajima Y Miyamoto Y Takigawa T A conserved non-reproductive GnRH system in chordates. PLoS ONE (2012) 7:e41955 10.1371/journal.pone.0041955 22848672 PMC3407064

[76] Rivero-Muller A Chou YY Ji I Lajic S Hanyaloglu AC Jonas K Rescue of defective G protein-coupled receptor function in vivo by intermolecular cooperation. Proc Natl Acad Sci USA (2010) 107:2319–24 10.1073/pnas.0906695106 20080658 PMC2836644

[77] Narumiya S Sugimoto Y Ushikubi F Prostanoid receptors: structures, properties, and functions. Physiol Rev (1999) 79:1193–226 10508233 10.1152/physrev.1999.79.4.1193

[78] Matsui M Miyamoto A Evaluation of ovarian blood flow by colour Doppler ultrasound: practical use for reproductive management in the cow. Vet J (2009) 181:232–40 10.1016/j.tvjl.2008.02.027 18693121

[79] Fujimori C Ogiwara K Hagiwara A Rajapakse S Kimura A Takahashi T Expression of cyclooxygenase-2 and prostaglandin receptor EP4b mRNA in the ovary of the medaka fish, *Oryzias latipes*: possible involvement in ovulation. Mol Cell Endocrinol (2011) 332:67–77 10.1016/j.mce.2010.09.015 20932877

[80] Takahashi T Fujimori C Hagiwara A Ogiwara K Recent advances in the understanding of teleost medaka ovulation: the roles of proteases and prostaglandins. Zool Sci (2013) 30:239–47 10.2108/zsj.30.239 23537233

[81] McGraw DW Mihlbachler KA Schwarb MR Rahman FF Small KM Almoosa KF Airway smooth muscle prostaglandin-EP1 receptors directly modulate beta2-adrenergic receptors within a unique heterodimeric complex. J Clin Invest (2006) 116:1400–9 10.1172/JCI25840 16670773 PMC1451203

